# NACore Amyloid Formation in the Presence of Phospholipids

**DOI:** 10.3389/fphys.2020.592117

**Published:** 2020-12-18

**Authors:** Jon Pallbo, Masayuki Imai, Luigi Gentile, Shin-ichi Takata, Ulf Olsson, Emma Sparr

**Affiliations:** ^1^Division of Physical Chemistry, Department of Chemistry, Lund University, Lund, Sweden; ^2^Department of Physics, Graduate School of Science, Tohoku University, Sendai, Japan; ^3^Department of Chemistry, University of Bari Aldo Moro, Bari, Italy; ^4^J-PARC Center, Japan Atomic Energy Agency, Tokai, Japan

**Keywords:** amyloids, peptides, NACore, fibrillation, aggregation, lipids, vesicles, bilayers

## Abstract

Amyloids are implicated in many diseases, and disruption of lipid membrane structures is considered as one possible mechanism of pathology. In this paper we investigate interactions between an aggregating peptide and phospholipid membranes, focusing on the nanometer-scale structures of the aggregates formed, as well as on the effect on the aggregation process. As a model system, we use the small amyloid-forming peptide named NACore, which is a fragment of the central region of the protein α-synuclein that is associated with Parkinson’s disease. We find that phospholipid vesicles readily associate with the amyloid fibril network in the form of highly distorted and trapped vesicles that also may wet the surface of the fibrils. This effect is most pronounced for model lipid systems containing only zwitterionic lipids. Fibrillation is found to be retarded by the presence of the vesicles. At the resolution of our measurements, which are based mainly on cryogenic transmission electron microscopy (cryo-TEM), X-ray scattering, and circular dichroism (CD) spectroscopy, we find that the resulting aggregates can be well fitted as linear combinations of peptide fibrils and phospholipid bilayers. There are no detectable effects on the cross-β packing of the peptide molecules in the fibrils, or on the thickness of the phospholipid bilayers. This suggests that while the peptide fibrils and lipid bilayers readily co-assemble on large length-scales, most of them still retain their separate structural identities on molecular length-scales. Comparison between this relatively simple model system and other amyloid systems might help distinguish aspects of amyloid-lipid interactions that are generic from aspects that are more protein specific. Finally, we briefly consider possible implications of the obtained results for *in-vivo* amyloid toxicity.

## Introduction

Aberrant protein folding and aggregation are closely related to many diseases. Amyloids are one type of aberrant protein aggregates made of stacked β-sheets that form highly ordered fibrillar structures. Amyloids are associated especially with diseases that cause neural degeneration such as Alzheimer’s and Parkinson’s diseases (Chiti and Dobson, 2017; Eisenberg and Sawaya, 2017). In addition to proteins, a large variety of other biomolecules exist in the biological environment. One type is lipids, such as phospholipids, which make up the basic structures of cellular membranes in the form of bilayers. Lipids constitute roughly 40% of the non-protein dry mass of mammalian cells (Alberts et al., 2008), and amyloid formation thus occurs in a lipid-rich environment. Lipids have been of interest in relation to amyloids because they have been found accumulated with the protein material in amyloid deposits in diseased tissues. Reports of lipids present in amyloid deposits can be found at least as far back as 1969 (Bonar et al., 1969). In that early study, X-ray scattering experiments suggested the presence of lipids in amyloid deposits isolated from diseased spleen tissue. Lipids have also been found together with amyloids in neural tissues, such as in the brains of people with Alzheimer’s or Parkinson’s disease (Gellermann et al., 2005; Kiskis et al., 2015; Kaya et al., 2017; Shahmoradian et al., 2019). Furthermore, *in vitro* studies have demonstrated co-localization and disruptive effects of amyloids on phospholipid bilayers (Janson et al., 1999; Butterfield and Lashuel, 2010; Zhang et al., 2014; Kollmer et al., 2016). It has also been shown that lipid membranes may strongly interfere with the aggregation process and affect the kinetics during amyloid formation (Gorbenko and Kinnunen, 2006; Galvagnion et al., 2015; Iyer and Claessens, 2019). For these and other reasons, the interplay between lipids and amyloid forming peptides and proteins is believed to potentially play a key role in the pathophysiology of amyloid associated diseases. This might be particularly significant in neurodegenerative disorders since the function of neural tissues is dependent on lipid structures in axons, dendrites, and synaptic vesicles.

In this report we have investigated amyloid formation by the amyloid model peptide NACore and how the formed aggregates are affected by the presence of phospholipids, from a structural perspective on nanometer length-scales. NACore is an eleven amino acid residue peptide from the so-called non-amyloid-β component (NAC) region of the Parkinson’s disease associated protein α-synuclein (68-GAVVTGVTAVA-78) (Bodles et al., 2001; Rodriguez et al., 2015; Pallbo et al., 2019). This peptide has previously shown cytotoxic effects on cells *in vitro* (Bodles et al., 2001; Rodriguez et al., 2015). The short model peptide is suitable for studies of physicochemical aspects of amyloid formation and can further bring insights into the role of the amyloid-forming NACore segment in relation to the remaining segments of the full length protein. We have studied the effect of varying the lipid composition in terms of headgroup charge and acyl chain saturation. The structures of the aggregates were analyzed with cryogenic transmission electron microscopy (cryo-TEM), small and wide angle X-ray scattering (SAXS, WAXS), and small angle neutron scattering (SANS), as well as circular dichroism (CD) spectroscopy. One question we address is how intimate the interaction of lipids and amyloids are on the molecular level. In other words, to what extent there is formation of new peptide-lipid-mixed structures that are distinct from both pure peptide fibrils and pure lipid bilayers. We believe that the results obtained here, with the relatively simple model systems outlined above, give important clues about the underlying physico-chemical mechanisms of phospholipid-amyloid interactions. In the end, this might help us to better understand the role of lipids in the pathophysiology of amyloid associated diseases.

## Materials and Methods

### NACore Peptide and Phospholipids

Lyophilized NACore peptide powder was purchased from Innovagen AB [GAVVTGVTAVA, 944 g/mol, trifluoroacetic acid (TFA) salt, >95% purity as specified by the supplier] and used as supplied. The concentrations specified in the report refer to the amount of lyophilized peptide powder used. Phospholipids were purchased from Avanti Polar Lipids Inc., United States in powder form and were used as supplied. The phospholipids used in the experiments were mixtures in various proportions of 1-palmitoyl-2-oleoyl-glycero-3-phosphocholine (POPC), 1-palmitoyl-2-oleoyl-sn-glycero-3-phospho-L-serine (POPS), 1,2-dimyristoyl-sn-glycero-3-phosphocholine (DMPC), and 1,2-dimyristoyl-sn-glycero-3-phospho-L-serine (DMPS).

### Phospholipid Vesicle Preparation

The desired phospholipids were dissolved in a 9:1 mixture of chloroform and methanol (volume ratio) at a total lipid concentration of 10 mM. The required amount of solution was then dried into a lipid film in a glass vial under a stream of nitrogen gas, followed by further drying in vacuum overnight. A sodium phosphate solution (∼ pH 7) was then added to the film. A dispersion of small vesicles was obtained by vortexing followed by tip sonication (Sonics & Materials, Inc., United States, Vibra-Cell VCX 130). The sonication time was 10–15 min for POPC:POPS 8:2 and DMPC:DMPS 8:2, and 30 min for POPC in on-off cycles of 5–10 s, at an amplitude of 50–60%. Debris from the sonication tip was pelleted by centrifugation at 16 000 Relative Centrifugal Force (RCF) for 5 min, and the resulting supernatant was used as the final phospholipid vesicle dispersion for the experiments. For POPC:POPS 8:2 and DMPC:DMPS 8:2, the vesicle suspension appeared clear to the eye under ambient light, but for POPC alone some turbidity remained despite the longer duration of tip sonication (presumably due to lack of electrostatic repulsion between the bilayers).

### Peptide Fibrillation Procedure

Lyophilized NACore peptide was dissolved at the desired concentration in 2 mM NaOH (pH 11.3). Fibrillation was induced by mixing the dissolved peptide with a sodium phosphate solution, which lowered the pH to about 7 and yielded a final sample with either 0.1 mg/ml peptide + 1 mM NaH_2_PO_4_ + 1 mM Na_2_HPO_4_ (for the cryo-TEM experiments), or 0.3 mg/ml peptide + 2 mM NaH_2_PO_4_ + 2 mM Na_2_HPO_4_ (for the X-ray scattering and CD measurements). The concentrations used differed because the different techniques required different amounts of material (and the experiments were initially carried out as parts of different data sets). When fibrillation was performed together with phospholipid, the vesicle dispersion was added together with the sodium phosphate solution as the pH was lowered. Approximately equimolar amounts of peptide and phospholipid molecules were present in the samples.

### Cryogenic Transmission Electron Microscopy (cryo-TEM)

Vesicles composed of either POPC:POPS 8:2 or POPC only were prepared, and peptide fibrillation was induced by a change in pH. After 4 h of quiescent incubation at room temperature, a small part of the sample was taken out, vitrified, and observed with a JEM-2200FS (JEOL) transmission electron microscope with an accelerating voltage of 200 kV and an in-column energy filter for zero-loss imaging (Omega Filters, India), as has previously been described (Pallbo et al., 2019). Images were recorded digitally using a TVIPS TemCam-F416 digital camera.

### Small and Wide Angle X-Ray Scattering (SAXS and WAXS)

The peptide alone and peptide-lipid samples were prepared as described above and then incubated quiescently over night at room temperature. The aggregated material was collected as a pellet by centrifugation for 3 h at 10 000 RCF at room temperature (resulting in a roughly 10 times increase in concentration of aggregated material, as estimated from the volume of the pellet). Part of the collected material was then placed in a glass capillary tube, and X-ray scattering was performed using a Ganesha 300XL system (SAXSLab) with a Cu ULD SL X-ray source (Xenocs) and a 2D PILATUS 300k detector (Dectris). The measurements were done in vacuum at room temperature unless stated otherwise, and a total of three detector distances were used (corresponding to different *q*-value ranges, from about 3⋅10^–2^ to 25 nm^–1^), with a total of 6 h of exposure time for each sample. Phospholipid vesicles incubated alone without the peptide were also investigated in a similar way, as well as water which was used as background. The scattering from the water was subtracted from the scattering curves of the samples before plotting. Fittings were performed in MATLAB by implementing standard form factor scattering models from SasView^1^, see Supplementary Table 1 for details. Polydispersity in the models was simulated by drawing 10^4^ values of the polydisperse parameter from a lognormal probability distribution, calculating the scattering profile for each value, and averaging of all the resulting curves.

### Circular Dichroism (CD) Measurements

Samples were prepared in the same way as for X-ray scattering, except that they were not concentrated by centrifugation. At different time points of the incubation, 300 μl of each sample was transferred a 1 mm path length quartz cuvette (Hellma, Germany, 110-QS), followed by measurement of the CD spectrum using a JASCO J-715 instrument at room temperature (two accumulations, scan rate of 20 nm/min, 2 s response time, and 2 nm band width, resulting in a HT voltage below 600 V at all wavelengths). The CD spectrum from the same cuvette filled with water was used as a reference and subtracted from the sample spectra before plotting.

## Results

We have previously reported that pH can be used to control the fibrillation of NACore (Pallbo et al., 2019). At high pH, the peptide has a net negative charge and a higher aqueous solubility than at lower pH closer to its isoelectric point (∼ pH 5.5). Fibrillation of dissolved NACore can thereby be induced by lowering the pH toward the isoelectric point. For the present experiments, we prepared the initial peptide solution at pH 11.3 (2 mM NaOH) and induced fibrillation by the addition of NaH_2_PO_4_ solutions to a final pH of about 7. We performed fibrillation both in the absence and presence of phospholipids. The phospholipids were either a mixture of 80 mol% POPC and 20 mol% POPS (POPC:POPS 8:2), POPC alone, or a mixture of 80 mol% DMPC and 20 mol% DMPS (DMPC:DMPS 8:2). In all samples that contained both peptide and phospholipids the amounts of peptide and lipids were approximately equimolar.

### Cryo-TEM Reveals Affinity Between NACore Fibrils and Phospholipid Vesicles

**FIGURE 1:**
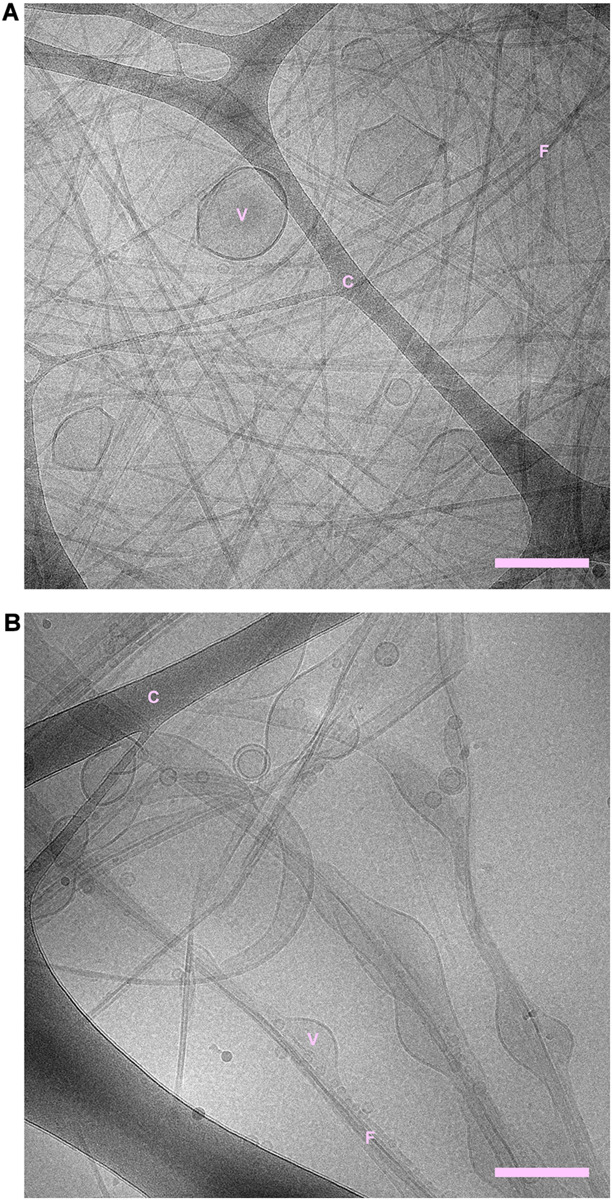
**(A)** NACore fibrillated in the presence of POPC:POPS vesicles. **(B)** NACore fibrillated in the presence of POPC vesicles. In both cases, highly distorted and trapped vesicles can be observed among the fibrils. There are more bilayer structures relative to the number of visible fibrils in the case of NACore with POPC than with POPC:POPS. “C” denotes the cryo-TEM carbon grid, “F” denotes fibrils, and “V” denotes distorted vesicles. Scale bars show 200 nm.

**FIGURE 2:**
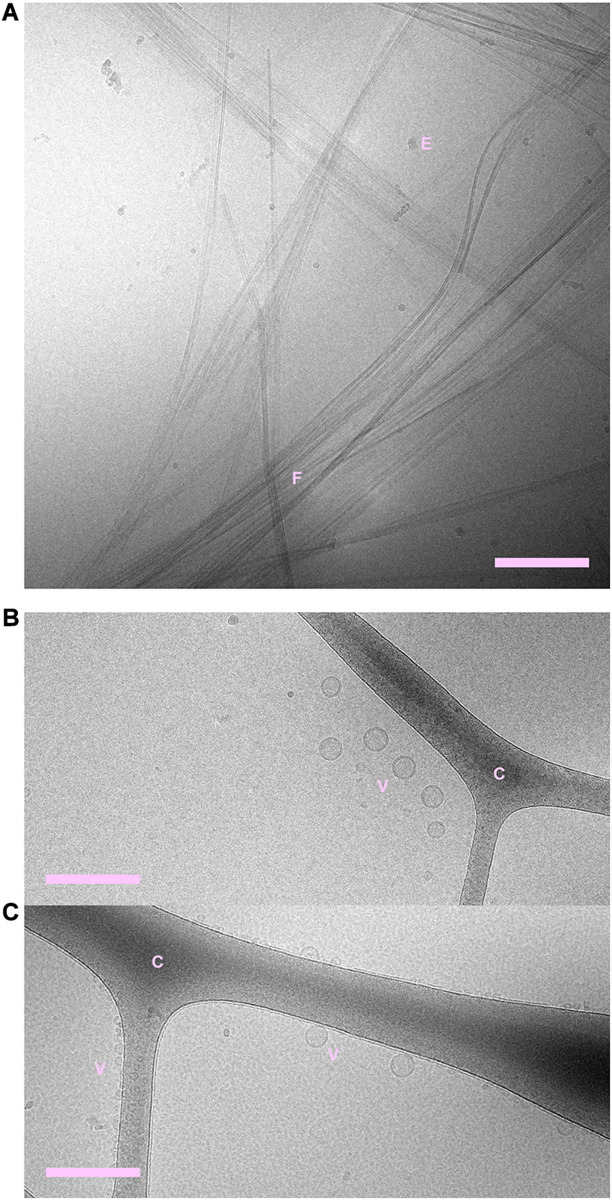
**(A)** NACore fibrillated in the absence of phospholipids. **(B)** POPC:POPS vesicles in the absence of NACore. **(C)** POPC vesicles in the absence of NACore. POPC vesicles were mainly found associated with the cryo-TEM carbon grid, rather than free in the solution. “C” denotes the cryo-TEM carbon grid, “F” denotes fibrils, “V” denotes vesicles, and “E” denotes suspected ethane contamination (not part of the sample itself). Scale bars show 200 nm.

Cryogenic transmission electron microscopy shows that fibrils are formed both in the absence and presence of phospholipid vesicles (Figures 1, 2 and Supplementary Figures 1–3). In the samples containing peptide and phospholipids, highly distorted vesicles can be observed interspersed among the fibrils (Figure 1 and Supplementary Figures 1, 2). In the case of POPC:POPS, many vesicles have highly distorted shapes as they are trapped in the fibril network, mainly as a consequence of the vesicle diameter being larger than the network mesh size. In the case of pure POPC, we observe lipid structures that strongly deviate from the originally sonicated vesicle dispersion. Many vesicles are much larger than in the original dispersion, presumably as a result of fusion events. Moreover, the shapes of these large vesicles deviate strongly from spheres and they appear to wet the fibril surfaces, indicative of an attractive fibril-bilayer interaction. It is also noted that substantially fewer fibrils are observed in the samples where NACore was aggregated in the presence of POPC vesicles (Figure 1B), compared to the sample with POPC:POPS vesicles (Figure 1A). While the distorted vesicles in both types of samples are associated with the fibrils, the bilayers of these distorted vesicles can still be rather clearly distinguished from the fibrils. In the samples containing POPC vesicles, some of them are multilamellar (Figure 1B) which is probably due to lack of electrostatic repulsion between the bilayers due to the zwitterionic nature of the glycero-3-phosphocholine (PC) head group (Scott et al., 2019). Multilamellar vesicles were observed also for POPC without NACore present (Supplementary Figure 4). In summary, the cryo-TEM images clearly show fibril-bilayer co-association, but they do not provide definite information about whether phospholipids are incorporated into the cross-β structure of the fibrils or if they are only adsorbed at the fibril surface.

### X-Ray Scattering of NACore Fibrillated in the Presence of POPC:POPS and POPC Vesicles

**FIGURE 3:**
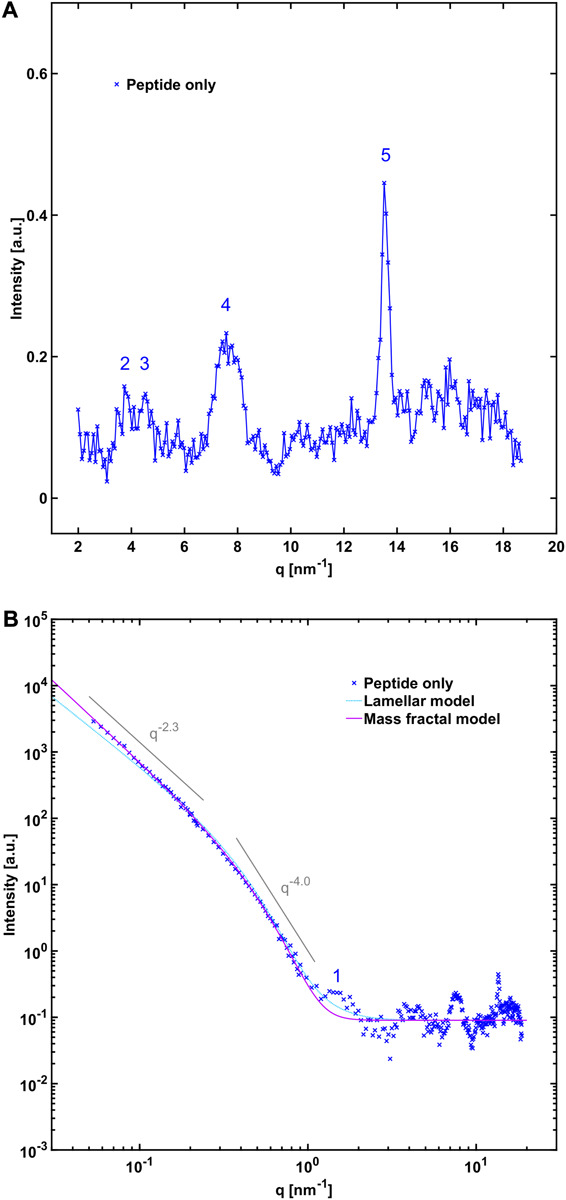
**(A)** WAXS and **(B)** SAXS profile of NACore fibrillated in the absence of phospholipids. The numbers denote crystal diffraction peaks corresponding to miller indices of 1 = [2, 0, 0], 2 = [2, 0, –1], 3 = [2, 0, 1], 4 = ([2, 0, –2], [0, 0, 2], [2, 0, 2]), and 5 = ([1, 1, –1], [1, 1, 1]) in the NACore crystal structure reported by Rodriguez et al. (PDB ID: 4RIL) (Rodriguez et al., 2015; Pallbo et al., 2019; see also Supplementary Figure 5). In **(B)** two theoretical fits are shown. One is an infinite lamellar model with a polydisperse average layer thickness of 6.5 nm (see Supplementary Table 1 for details). The other one is a mass fractal model with polydisperse average building block radius of 4.3 nm (see Supplementary Table 1).

In order to gain further insight into the structure of the fibrils formed when NACore is incubated in the presence of phospholipid vesicles at smaller length scales, we performed small and wide angle X-ray scattering experiments (SAXS and WAXS). Below, we first describe the X-ray scattering data for samples composed either of peptide fibrils or lipid vesicles by themselves, and thereafter we analyze the aggregates formed when peptide has been left to aggregate in the presence of vesicles with different composition. Figure 3 shows WAXS and combined SAXS/WAXS data for aggregates formed by peptide alone. Here, the scattered intensity I(q) is plotted against the scattering vector q. These X-ray scattering patterns are in close agreement with previously reported data for NACore peptide fibrils formed under similar conditions, showing a characteristic power law scattering pattern at low *q*-values, and several Bragg peaks at higher *q*-values (Pallbo et al., 2019). The two main peaks occurring at *q* = 13.6 nm^–1^ and 7.6 nm^–1^, are associated with the β-strand separation of 0.46 nm and the β-sheet lamination distance of 0.82 nm (PDB ID: 4RIL) (Rodriguez et al., 2015). The SAXS pattern shows a power law dependence of about −2.3 at low *q*-values, with a cross-over to a power law of −4 (Porod regime) occurring around a *q*-value of 0.3 nm^–1^ (Figure 3B). The latter being consistent with an average cross section radius of the fibrils of circa 4 nm, which in turn is consistent with the cryo-TEM images. For long thin fibrils we would expect to see I(q)∼q^–1^ at lower *q*-values (Lindner and Zemb, 2002). The fact that we see a much steeper decay, q^–2.3^, indicates attractive fibril–fibril interactions resulting in an inhomogeneous distribution of fibrils forming clusters. The value 2.3 can be interpreted as a mass fractal dimension of the clusters (Solomon and Spicer, 2010).

**FIGURE 4:**
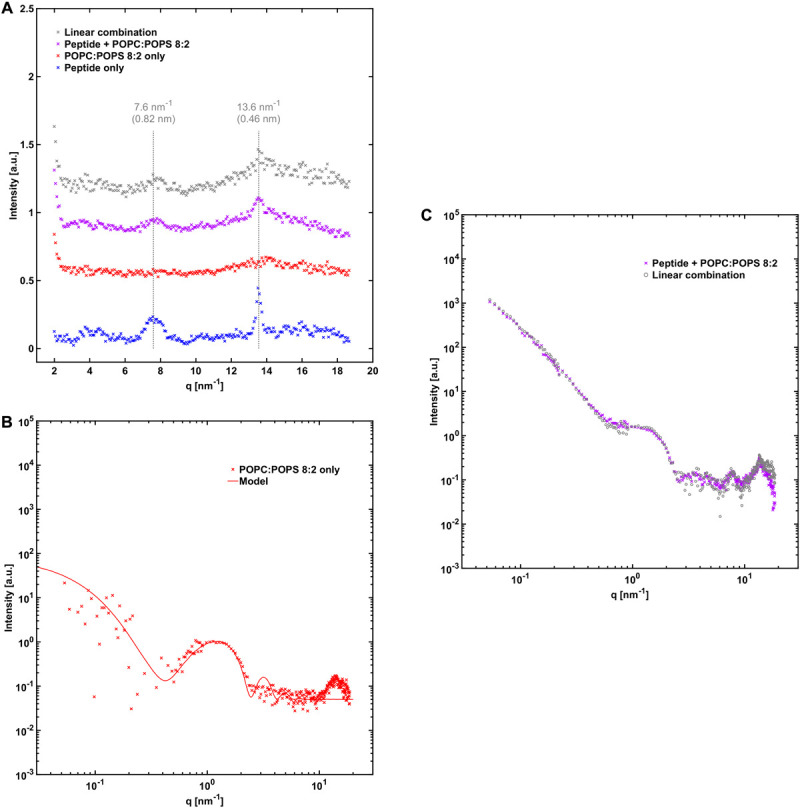
NACore together with POPC:POPS. **(A)** WAXS profile of NACore with POPC:POPS, POPC:POPS by itself, and the peptide by itself. The position of the two main diffraction peaks are indicated with dashed lines. For comparison, the gray curve shows the expected scattering for the same linear combination as in **(C)**. **(B)** SAXS profile of 2.4 mM POPC:POPS without the peptide, together with a simple unilamellar vesicle model fit (see Supplementary Table 1). **(C)** SAXS profile of co-incubated NACore and POPC:POPS, together with a linear combination of scattering from peptide fibrils (Figure 3B) and POPC:POPS vesicles **(B)** (0.40⋅fibril + 1.55⋅vesicles).

**FIGURE 5:**
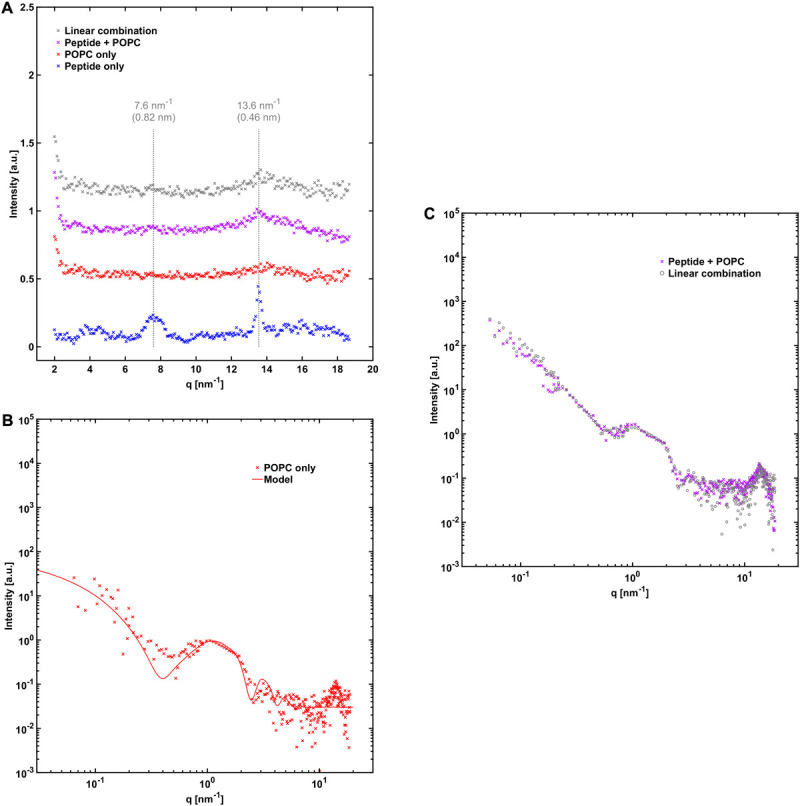
NACore together with POPC. **(A)** WAXS profiles similar to those in Figure 4A, except that POPC was used instead of POPC:POPS. **(B)** 2.4 mM POPC without the peptide together with a simple vesicle model fit with 90% unilamellar and 10% bilamellar vesicles (see Supplementary Table 1). **(C)** SAXS profile of NACore co-incubated with POPC, together with a linear combination of scattering from peptide fibrils (Figure 3B) and POPC vesicles **(B)** (0.15⋅fibrils + 1.40⋅vesicles).

The average X-ray scattering length densities (electron densities) of the phospholipid bilayers investigated are close to that of water (approximately 9.8⋅10^–6^ Å^–2^ and 9.5⋅10^–6^ Å^–2^ for the bilayers and water, respectively), which means that the vesicles scatter very little at lower *q*-values. Consequently, the size and shape of the vesicles cannot be easily determined using SAXS. However, because of the relatively strong difference in scattering length density between the phospholipid headgroups and the acyl chains (13.5⋅10^–6^ Å^–2^ and 8.0⋅10^–6^ Å^–2^), there is a significant electron density variation and hence contrast in the direction normal to the bilayer. This allows some features of the bilayers to be determined, such as the thickness and overall internal structure. We can see from the scattering pattern of POPC:POPS vesicles that it fits well with the scattering pattern of a single bilayer with a headgroup-to-headgroup distance of about 3.8 nm (Figure 4B), consistent with expectations from the literature (Kucerka et al., 2011; Eicher et al., 2017; Scott et al., 2019). In the case of vesicles composed of POPC only, we can fit them with the scattering of bilayers with the same headgroup-to-headgroup thickness as for POPC:POPS (Figure 5B).

A comparison of the X-ray scattering pattern between peptide fibrillated in the absence and presence of phospholipid vesicles provides information about how the structures are affected at nanometer length-scales. We are here interested in knowing whether the phospholipids from the vesicles are incorporated into the cross-β structure of the fibrils, or whether vesicles are just associated with the exterior fibril surfaces as, for example, adsorbed vesicles or deposited lipid bilayers. One way to obtain such information is to look at the positions of the Bragg peaks at high *q*-values, which arise from the crystal-like cross-β molecular packing of the peptide molecules within the fibrils, and how they are affected by the presence of phospholipids. To probe structural changes on slightly longer length-scales, we investigated whether the X-ray scattering pattern can be fitted as a linear combination of peptide fibrils alone and phospholipid bilayers alone, which should be the case if most of the phospholipid bilayers and fibrils exist as independent structures. If a linear combination of the individual components agrees poorly with the data, it would be an indication of new structures formed, or of substantial inter-species interactions.

From the X-ray scattering experiments we find that the positions of the two major cross-β associated Bragg peaks at 7.6 nm^–1^ and 13.6 nm^–1^ (corresponding to 0.82 nm and 0.46 nm periodicities in real space) are not markedly affected by the presence of POPC:POPS bilayers (Figure 4A). However, we note that the intensity of the peaks is substantially lower in the co-incubated sample, relative to peptide alone. This is consistent with a reduced amount of peptide fibrils in the presence of lipids. This will be discussed in more detail below. In the case of NACore together with vesicles composed of POPC only, the position of the peak at 13.6 nm^–1^ was unchanged, whereas the signal was too weak to resolve the peak at 7.6 nm^–1^ (Figure 5A). Furthermore, the X-ray scattering pattern of co-incubated peptide and POPC:POPS or POPC vesicles can be very well fitted as a linear combination of the scattering pattern of the two separate components (Figures 4C, 5C). This suggests that the co-incubation with phospholipids does not considerably alter the bulk of the structure of the fibrils on nanometer length scales. It also indicates that the bulk of the POPC:POPS and POPC bilayers are not considerably altered by the presence of the NACore peptide in terms of bilayer thickness and overall internal structure. However, these X-ray scattering experiments are not very sensitive to changes in features such as the size or shape of the vesicles. We finally conclude that most of the phospholipids that were present in the initial co-incubation suspensions were co-pelleted together with the fibrils in the centrifugation step, as judged by the bilayer contribution in the linear combinations used to fit the scattering data for the samples containing both peptide and lipid. This was the case both for NACore aggregated together with POPC:POPS vesicles and POPC vesicles (Figures 4, 5). The vesicles alone are not pelleted by the centrifugation step when they are small and unilamellar. This observation thus gives further support to the conclusion based on the cryo-TEM experiments that vesicles become attached to the fibrils.

### Effect of Phospholipids on the Amount of Fibrils

**FIGURE 6:**
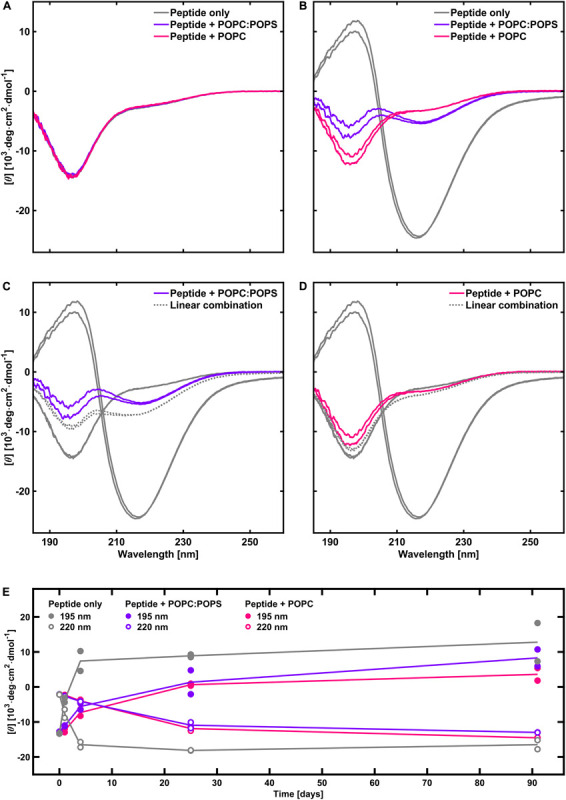
CD spectra of peptide in the absence and presence of phospholipids, with duplicate samples for each system. **(A)** Initial time point. **(B)** After 1 day. **(C)** Estimation of the extent of fibrillation in the presence POPC:POPS (gray dotted line) relative to peptide fibrillated alone, based on a linear combination of the spectra for peptide alone at *t* = 0 and at *t* = 1 day (80% of the spectrum at *t* = 0 plus 20% of the spectrum at *t* = 1 day). **(D)** Same as **(C)** but with POPC instead of POPC:POPS. The linear combination shows 95% of the peptide alone spectrum at *t* = 0 plus 5% of the spectrum at *t* = 1 day. **(E)** Time evolution of the CD intensity at 195 and 220 nm, with and without phospholipid vesicles (based on a different peptide batch). It shows that the fibrillation continues to increase after 1 day in the presence of phospholipids. Phospholipids thus have an inhibiting effect on the rate of fibrillation.

One observation from the X-ray scattering data is that the scattering intensity at low *q*-values was substantially lower in the presence of phospholipid vesicles than for peptide fibrillated alone. This was especially true in the case when the peptide was fibrillated in the presence of POPC vesicles, where the intensity is almost an order of magnitude lower compared to the sample with peptide alone (15% of the intensity, Figure 5, compared to 40% for POPC:POPS, Figure 4). The difference between the case with POPC and POPC:POPS was also indicated by fewer fibrils found in the cryo-TEM images in the presence of POPC vesicles (Figure 1). This indicates that after 1 day of aggregation, the samples with phospholipids contain lower amounts of fibrils. To further investigate these differences, we performed CD measurement on peptide fibrillated in the presence and absence of POPC:POPS or POPC vesicles at various time points of incubation (Figure 6). The CD measurements give information about the secondary structure of the peptide and can be used to monitor the peptide fibrillation process (Kelly et al., 2005; Pallbo et al., 2019). Initially, the peptide has a disordered structure as judged from the CD spectra. The spectra for the initial state (*t* = 0) are essentially identical for all three samples (Figure 6A), implying that the structure of the initial unfibrillated peptide is the same in all cases, and that the phospholipids did not contribute with a CD signal of their own. After fibrillation has been induced, the CD spectra transform and reveal the presence of β-sheet-rich structures, which constitute the building blocks of the NACore amyloid fibrils (Figure 6B). Consistent with the X-ray scattering data, the CD measurements confirmed a rather substantial reduction of β-sheet content in the presence of both types of phospholipid vesicles, with the most prominent difference in the presence of POPC vesicles. This supports the conclusion that the differences in intensity observed in the X-ray scattering experiments were due to actual differences in amounts of fibrillated material. Another implication from the CD and X-ray measurements is that although samples were prepared with an approximately equimolar amount of total peptide and phospholipid molecules at *t* = 0, the molar fraction of fibrillated peptide relative to phospholipid was probably substantially lower than 1 at the time point of the X-ray scattering measurements (roughly 0.2–0.4 for POPC:POPS and 0.05–0.15 for POPC).

### Fibrillation in the Presence of DMPC:DMPS Vesicles

**FIGURE 7:**
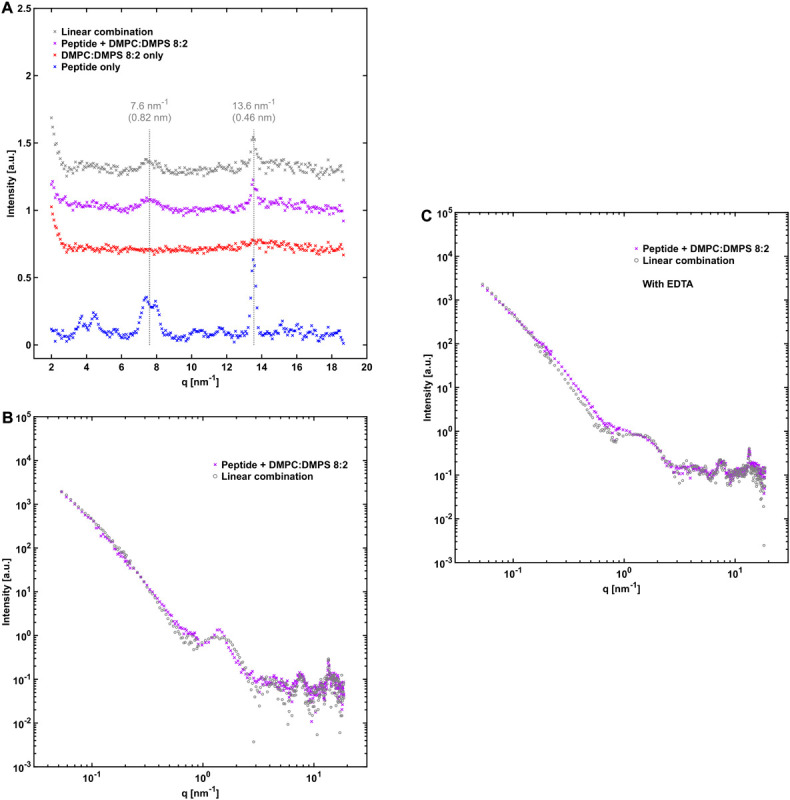
NACore together with DMPC:DMPS. **(A)** WAXS profiles similar to those in Figures 4A, 5A, but with DMPC:DMPS as the lipid system. **(B)** SAXS profile of NACore co-incubated with DMPC:DMPS, together with a linear combination of scattering from fibrils and vesicles (0.30 fibrils + 1.20⋅vesicles). A sharp peak at *q* = 1.4 nm^–1^ is present in the scattering profile of the co-incubated sample. **(C)** SAXS profile of NACore co-incubated with DMPC:DMPS, together with a linear combination of scattering from fibrils and vesicles (0.70⋅fibrils + 0.90⋅vesicles), with 0.1 mM added EDTA. The sharp peak at *q* = 1.4 nm^–1^ is not present. For all experiments with this lipid system incubation and measurements were done at 37°C instead of at room temperature.

For further characterization of peptide-phospholipid interactions we performed X-ray scattering, contrast variation SANS, and cryo-TEM analysis for NACore that was aggregated together with vesicles composed of DMPC:DMPS, in both hydrogenated and deuterated form. DMPC and DMPS were used instead of POPC and POPS because the latter are not readily available in fully deuterated form. The WAXS pattern for the peptide together with DMPC:DMPS gave similar results as with the other lipid compositions in that the positions of the major cross-β associated Bragg peaks were not altered by the co-incubation with the lipids (Figure 7A). However, the scattering profile at lower *q*-values revealed a difference compared to the results with POPC:POPS and POPC. A small peak was present at 1.4 nm^–1^ in the scattering profile, which could not be fitted as a linear combination of the scattering from the separate components (Figure 7B). Further analysis based on neutron scattering and cryo-TEM data revealed that this was due to the presence of long multilamellar helical tubes in the samples (Supplementary Section 1). The peak at *q* = 1.4 nm^–1^ arise from the periodic spacing of *d* = 4.4 nm in the multi-lamellar tube wall. Similar structures have been reported before for other lipids (Fuhrhop and Helfrich, 1993), and for lipids with sn-glycero-3-phospho-L-serine (PS) head groups in the presence of divalent metal ions (Ca^2+^ in particular) which are then referred to as cochelates (Papahadjopoulos et al., 1975; Nagarsekar et al., 2016). We believe this is also the structures observed in our samples with DMPC:DMPS. Ca^2+^ can leach from some types of glassware under high pH conditions, and we found small amounts of Ca^2+^ that appeared to be present along with the lyophilized peptide powder (about 16 μM, see Supplementary Section 2). Indeed, when the X-ray scattering experiments were repeated in the presence of 0.1 mM added ethylenediaminetetraacetic acid (EDTA), the sharp peak at 1.4 nm^–1^ was absent and the resulting scattering curve more closely resembled those obtained with POPC:POPS and POPC. However, the fit obtained from a linear combination of the components was still slightly worse than for the other lipid systems (Figure 7C). We note that these samples were prepared and measured at 37°C instead of room temperature, due to the relatively high hydrocarbon chain melting point of DMPC:DMPS.

## Discussion

Here we have studied the 11 amino acid residue peptide NACore that is a fragment from the central NAC region of the 140 amino acid residue protein α-synuclein. NACore can form amyloid fibrils by itself without the rest of the full-length α-synuclein protein (Rodriguez et al., 2015; Pallbo et al., 2019), indicating that the driving forces for amyloid formation are still present for this small peptide fragment. However, other properties will of course differ between the NACore peptide fragment and the full-length α-synuclein protein. For example, NACore does not include segments of α-synuclein that are considered crucial for some of its interaction with lipid membranes. Monomeric α-synuclein has a strong affinity for anionic phospholipid bilayers. The N-terminal region of α-synuclein (from approximately residue 1 to 100) folds into an α-helix when it adsorbs to such anionic membranes. The C-terminal region, on the other hand, remains unstructured in the membrane-bound state and extends from the membrane surface (Pfefferkorn et al., 2012). An adsorbed layer of α-synuclein on anionic lipid membranes might be crucial for triggering α-synuclein amyloid formation (Galvagnion et al., 2015; Gaspar et al., 2020). Similarities and differences between the lipid interactions of the short NACore peptide fragment studied in this report, full length α-synuclein, and other proteins might highlight which features of the interactions are likely to be generic for most amyloid forming peptides and proteins, and which might be more protein specific. We have focused on the effects of phospholipids on the cross-β packing in the fibrils, lipid-peptide co-assembly, as well as on effects of lipids on the fibrillation process. Below these aspects will be discussed separately. Finally, the potential *in-vivo* significance will be considered.

### Effects on Cross-β Packing and Fibril Morphology

One finding from the present study is that although there is lipid-protein co-assembly at larger length scales, the presence of phospholipids has no detectable effect on the cross-β organization in the amyloid fibrils at the resolution of our measurements. This suggests that the peptide-peptide interaction in the crystal-like fibrils is stronger than the peptide-lipid interaction, and that is perhaps not very surprising considering the typical observation of low solubility of solutes in crystalline solids. For example, crystallization is commonly used as a way of purifying chemicals from a mixture of substances (Mullin, 2001). The observation of no effect on the position of the main cross-β associated Bragg peaks from the peptide fibrils is also consistent with the very early report of amyloid lipid co-aggregation in diseased spleen tissue that was mentioned in the introduction (Bonar et al., 1969). In wide angle X-ray scattering they observed peaks typical of the cross-β structure found in amyloids, together with additional peaks that were likely due to lipid material. Delipidation of the sample removed the extra peaks but left the cross-β-associated peaks of the amyloid material unaffected, suggesting that the internal structure of the amyloid fibrils remained the same. However, it cannot be excluded that differences could be found by more sensitive measurements than those reported here. We also note that the fibril morphology, such as cross section dimensions, were not significantly altered when fibrillation occurred in the presence of lipid vesicles, compared with fibrillation in the absence of lipid. The SAXS pattern of mixed systems could be well described by a superposition of pure fibril and vesicle scattering. This implies that whatever the lipid-fibril interaction, final fibril morphology is not substantially affected.

### Effect on Lipid Self-Assembly

The bilayer cross section form factors of POPC:POPS and pure POPC lipid systems were not affected by the presence and fibrillation of the NACore peptide (Figures 4C, 5C), as data from the mixed fibril-lipid systems could be described by a superposition of the scattering patterns observed in pure lipid and peptide samples, respectively. In fact, the SAXS cross section form factor of lipid bilayers is generally sensitive to variations in bilayer thickness and area per lipid molecule (head group area density) (Pabst et al., 2000; Saveyn et al., 2009). From this we may conclude that the lipid bilayers remain essentially intact in the presence of peptide. Thus, there are no indications of significant solubilization of peptide monomers in the bilayer membranes. Similar conclusions can be drawn for the case of DMPC:DMPS.

### Co-aggregation With Lipids

We can distinguish between association of lipids and peptide in the monomeric and in the fibrillar state. In the present study we found a clear affinity between peptide fibrils and phospholipid vesicles. The lipid bilayers of the vesicles seem to have high affinity for the fibril surface, leading to vesicle attachment and deformations. Similar behavior has been demonstrated with amyloid fibrils made of proteins such as β2-microglobulin (Milanesi et al., 2012) and α-synuclein when aggregated with an excess of anionic vesicles (Hellstrand et al., 2013). Complete rupture of vesicles was observed for some lipid compositions and lipid-protein ratios (Hellstrand et al., 2013; Galvagnion et al., 2019). Some studies have also identified interaction with amyloid fibril ends as being especially disruptive for phospholipid vesicles (Milanesi et al., 2012; Kollmer et al., 2016). However, due to the long nature of the fibrils in our system, meaning low occurrence of ends, we could not readily assess that mode of interaction for NACore. The observed co-existence of fibrils and surface-associated vesicles is consistent with the X-ray scattering data (Figures 4, 5) since the SAXS profiles of the fibrillated samples containing both peptide and lipid could be fitted as linear combinations of fibrils and bilayers.

The fibrillation experiments were performed by dropping the pH from 11 to 7, where the hydrophobic peptide has essentially zero net charge. The aqueous solubility of the peptide is thereby drastically reduced, the solution becomes supersaturated, and the peptide precipitates out of solution. The self-assembly is driven by hydrophobic interactions. The fibrillar aggregates formed are also hydrophobic, and further aggregate into clusters as seen by the power law dependence I(q)∼q^–2.3^ at lower *q*-values. Thus, we may in principle expect hydrophobic attraction both between fibril and bilayer and between peptide monomer and bilayer. The different faces of the NACore amyloid fibrils are expected to have different surface properties. Based on the known crystal structure of NACore (Rodriguez et al., 2015; Pallbo et al., 2019) two faces of the fibrils have no charges and consist mostly of non-polar amino acid side chains. The other two exposed sides consist of oppositely charged N- and C-terminals of the peptide, stacked next to each other. As the fibrils have a net zero charge, hydrophobic interactions are most likely the dominating attractive interaction between the fibrils and lipid bilayers. Previous studies of co-assemblies of α-synuclein and phospholipids have demonstrated reduced molecular dynamics in the associated lipid molecules both in the headgroups and in hydrocarbon chain segments close to the headgroups (Hellstrand et al., 2013; Galvagnion et al., 2019).

Next, we consider the possibility of peptide-lipid co-assembly already in the initial stage of the process at about *t* = 0, when the peptide is still in monomer form. If a large proportion of monomeric NACore peptide is adsorbed to the phospholipid bilayers already at *t* = 0, we can tell from the CD spectra that the adsorption in such a case is not associated with any detectable conformation change. The CD spectra at short times are essentially identical in the presence and in the absence of lipid (Figure 6A). From a colloidal point of view, it is not surprising if the degree of association between the peptide and the lipid bilayer would increase as the peptide particles grow larger, such as when they form fibrils, because as the particles grow larger the strength of the interactions per particle increases. It is thus possible that the affinity between monomeric NACore peptide and phospholipid vesicles is rather weak, while the affinity is very strong between the fibrils and the vesicles.

### Effects on the Fibrillation Process

An important finding from the CD (Figure 6) and X-ray scattering experiments is that the presence of phospholipids reduces the amount of fibrils formed after 1 day of incubation. This is observed both for the system containing POPC:POPS vesicles and POPC vesicles, with the strongest reduction observed for the latter system. As shown by the long time CD study (Figure 6E), this can be explained by a slowing down of the fibrillation kinetics rather than just a reduction of the final steady-state amounts of fibrils formed. This is because the extents of β-sheet signal have approached each other for all the samples at the longest time point relative to the earliest time points. This behavior is different from that observed with full-length α-synuclein, where fibrillation instead is accelerated by the presence of anionic vesicles for certain protein-to-lipid ratios (Galvagnion et al., 2015; Gaspar et al., 2018). One difference between these systems is that monomeric α-synuclein adsorbs to anionic membranes where it adopts a partial α-helical structure, while there are no signs of conformational changes for the NACore peptide when exposed to either zwitterionic or anionic vesicles. In quiescent conditions and pure buffer solutions, monomeric α-synuclein does not readily nucleate within time frames of several days. Lipid bilayers are thought to act as sites of primary nucleation (Galvagnion et al., 2015; Gaspar et al., 2018). In the kind of experiments with NACore performed here, the nucleation of fibrils occurs spontaneously also in the absence of phospholipid bilayers, and the rate of fibril formation is instead decreased due to some interaction with the bilayers. Assuming that extinction coefficients of the peptide UV absorption is unaffected by the presence of lipid, then we can use the CD signal to estimate the amounts of fibrils formed. For example, we can follow the time evolution of the CD signal at 220 nm, as presented in Figure 6E. From comparing the time evolutions in the presence of lipid with that in absence of lipid, it is revealed that the presence of lipid reduces the fibrillation rate by more than an order of magnitude. On the other hand, the amounts of fibrils that have formed at long time when steady state has been achieved, is only reduced by 10–20%, if at all.

Both lipid-fibril and lipid-monomer interactions may affect the fibrillation rate. Lipids may adsorb onto the growing fibrils, obstructing the attachment of peptide monomers to the aggregates. Such a mechanism has been observed for other systems, such as for the inhibiting effect of the polymer polyvinylpyrrolidone (PVP) on the crystal growth rate of bicalutamide (Lindfors et al., 2008). In that study, PVP was found to adsorb to the particle surfaces and thereby reduce the rate of crystal growth. PVP, however, was found not to affect the initial nucleation rate. When, in a nucleation and growth process, the growth rate is reduced without affecting the nucleation rate, this is expected to lead to smaller particles but larger in number. As these peptide fibrils in general grow to become very long, it is not possible to clearly say from the cryo-TEM images and SAXS patterns that they are significantly shorter in the presence of lipid. It is at the same time clear that we do not see evidence for a large fraction of relatively small fibrils (<100 nm). The fibrils observed in cryo-TEM (Figures 1, 2 and Supplementary Figures 1–3) are all very long also in the presence of lipid.

A reduced fibrillation rate may also result from peptide monomers being solubilized in, or adsorbed to lipid bilayers, thereby reducing the effective supersaturation. The peptide fibrillation can be seen as the precipitation of a solid crystalline phase. In the aqueous bulk monomer solution (*m*), the (monomer) peptide chemical potential can be written as $\mu_{m}=\mu_{m}^{0}+k_{B}\,T\,\ln c_{m}$, where $\mu_{m}^{0}$ is the concentration independent part and *c*_*m*_ is the bulk monomer concentration. *k_*B*_T* is the thermal energy with *k*_*B*_ being Boltzmann’s constant and *T* the absolute temperature. In the solid fibril phase (*f*), the corresponding peptide chemical potential can be considered as constant, $\mu_{f}=\mu_{f}^{0}$. The equilibrium monomer concentration, *S*, coexisting with the fibril phase, i.e., the monomer solubility, is then given by $S=\exp\left\{\left(\mu_{f}^{0}-\mu_{m}^{0}\right)\,/\,k_{B}\,T\right\}$. Fibrils are nucleated from supersaturated solutions where *c*_*m*_ > *S*, and the rate of nucleation, and also aggregate growth, is usually a strong function of the degree of supersaturation, *c*_*m*_/*S* (Kashchiev, 2000), also in the case of peptide fibrillation. The lipid bilayers can be seen as a third phase. If monomers have affinity to that phase, being adsorbed or solubilized, *c*_*m*_ is reduced. If the peptides would dissolve mostly within the membrane, we would expect that to involve a change in secondary structure, e.g., to α-helix, with hydrogen bonding to water being replaced by intramolecular hydrogen bonding. No sign of this is observed in the CD spectrum at short times. Nor do we see by SAXS any change in the lipid bilayer form factor in the presence of peptide. Therefore, if the peptide monomers have an affinity for the lipid bilayers, they are likely adsorbed within the headgroup region, still hydrated and with random coil conformation. In the case of adsorption, we expect the adsorbed amount, *Γ*, to be proportional to *c*_*m*_, unless we are close to saturation (Evans and Wennerström, 1999). For reversible monomer adsorption, this mechanism may lead to a significant reduction of nucleation and growth rates, with only a minor decrease in final fibril concentration, as the adsorbed monomers still act as a reservoir due to reversibility, decreasing *Γ* as *c*_*m*_ decreases. If the adsorption is saturated initially, *Γ*∼*c*_*m*_ may be achieved at later times.

In the present experiments, the peptide-to-lipid molar ratio was close to one. Hence, if one peptide monomer adsorbs to every second lipid molecule, that is, to every lipid molecule of the outer monolayers of the vesicle bilayers, then *c*_*m*_ is initially reduced by approximately one half. Whether this mechanism may quantitatively explain the observed kinetics remains to be shown. Discriminating whether peptide monomer-lipid association is occurring requires further studies. This may be done by measuring fibrillation kinetics as a function of peptide concentration and as a function of the peptide-lipid molar ratio.

### *In-vivo* Significance

One important part of the physiology of cells and neural tissues is vesicle transport. Even without total disruption of vesicles, hindering of vesicle transport could be a mechanism of toxicity for cells (Auluck et al., 2010). In our experiments we have seen clear evidence of vesicles being trapped in fibril networks. Similar effects of organelles and lipid structures being entangled in fibrils have been seen in other studies (Auluck et al., 2010; Shahmoradian et al., 2019). Misfolding and fibrillation of proteins results in long colloidal objects with a lot of potentially very sticky surface area. When such sticky surfaces in the cellular environment lead to capture and entanglement of vesicles and organelles it could thus potential lead to cellular toxicity.

## Data Availability Statement

The raw data supporting the conclusions of this article will be made available by the authors, without undue reservation.

## Author Contributions

JP, UO, and ES designed the research and wrote the manuscript with input from MI, LG, and ST. JP, LG, MI, and ST performed neutron scattering experiments. JP performed all other experiments except the cryo-TEM imaging and elemental analysis and analyzed the data with input from UO and ES. All authors approved the manuscript before submission.

## Conflict of Interest

The authors declare that the research was conducted in the absence of any commercial or financial relationships that could be construed as a potential conflict of interest.

## Footnotes

1 http://www.sasview.org/
