# Supplementary material for: NACore Amyloid Formation in the Presence of Phospholipids
Source: Front Physiol. 2020 Dec 18;11:592117. doi: 10.3389/fphys.2020.592117 (PMC7775532; doi:10.3389/fphys.2020.592117)
Supplement: Supplementary file 1 [file Data_Sheet_1.PDF]

# Supplementary Material

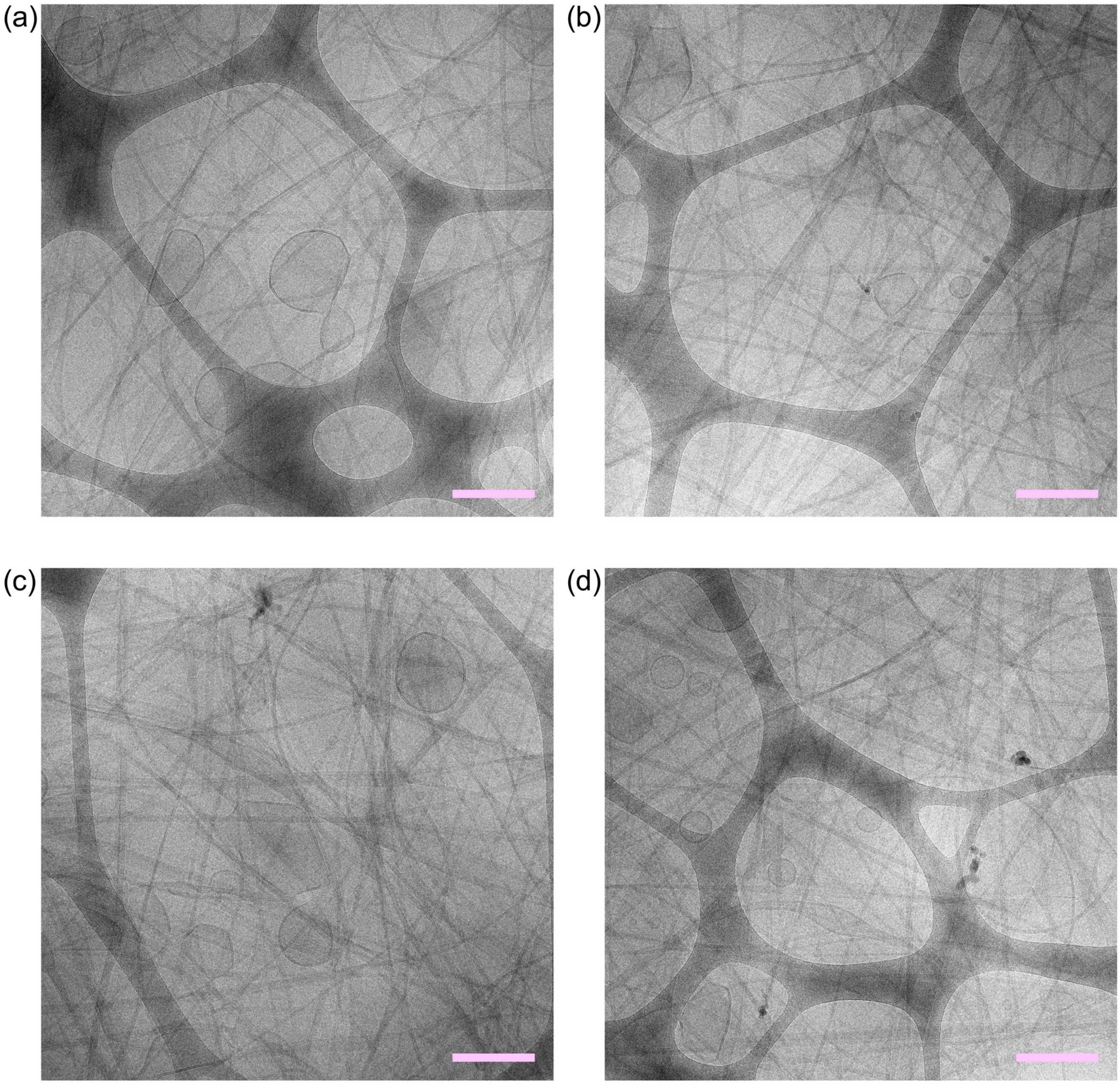


**Figure S1.** (a) To (d), additional cryo-TEM images of NACore together with POPC:POPS vesicles from the same sample as in Figure 1a in the main manuscript. The scale bars show 200 nm.

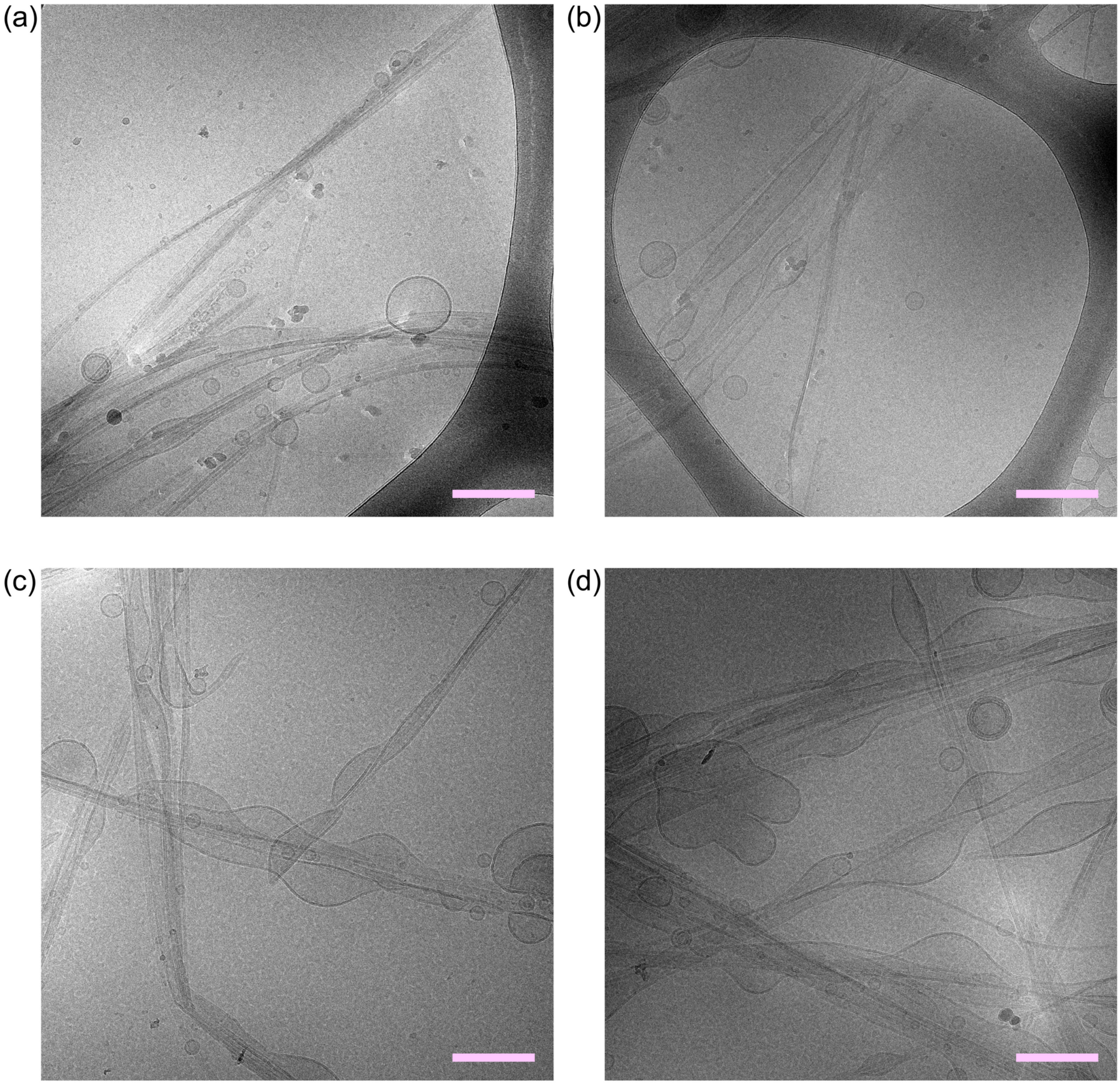


**Figure S2.** (a) To (d), additional cryo-TEM images of NACore together with POPC vesicles from the same sample as in Figure 1b in the main manuscript. The scale bars show 200 nm.

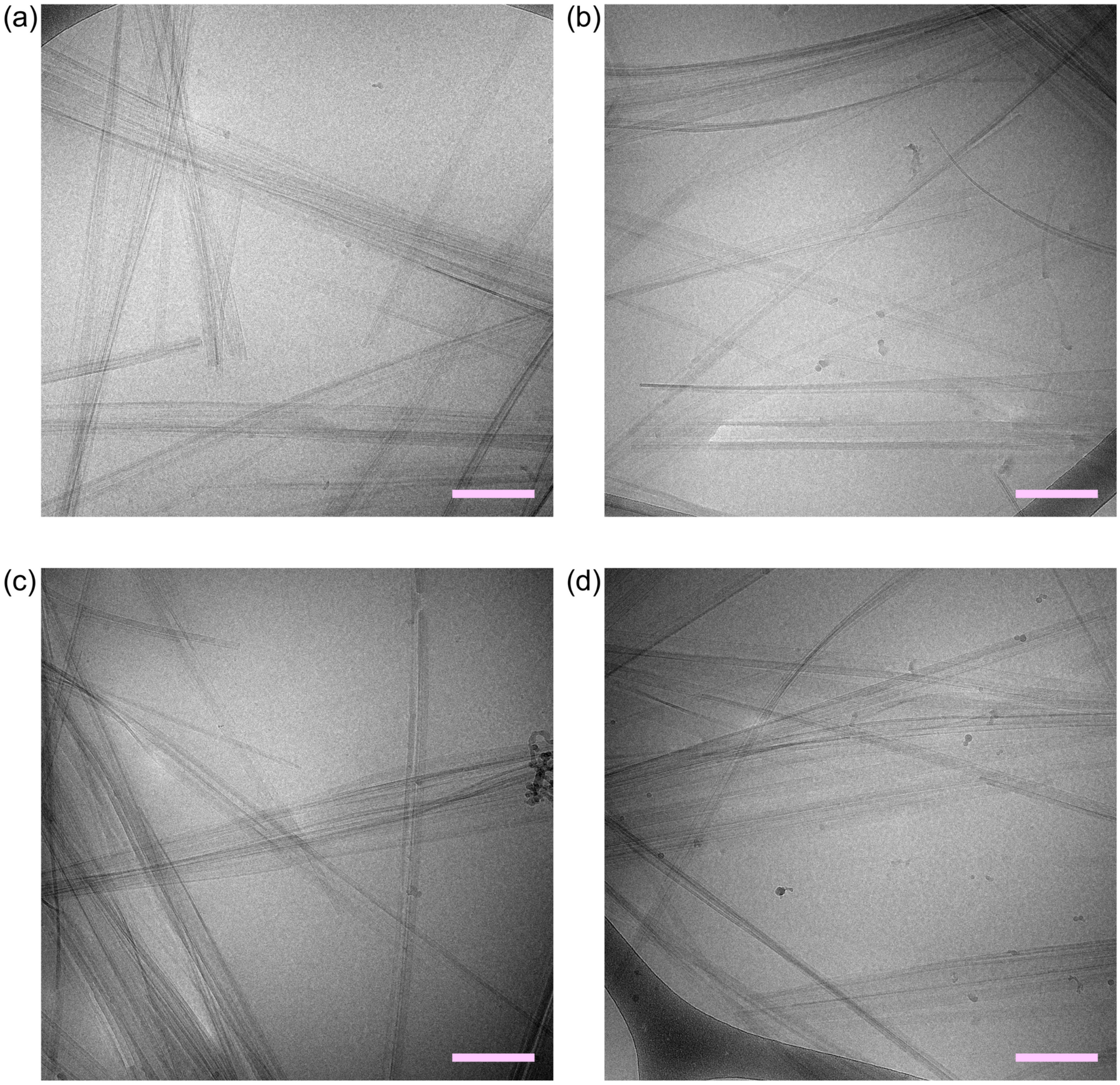


**Figure S3.** (a) To (d), additional cryo-TEM images of NACore without vesicles from the same sample as in Figure 2a in the main manuscript. The scale bars show 200 nm.

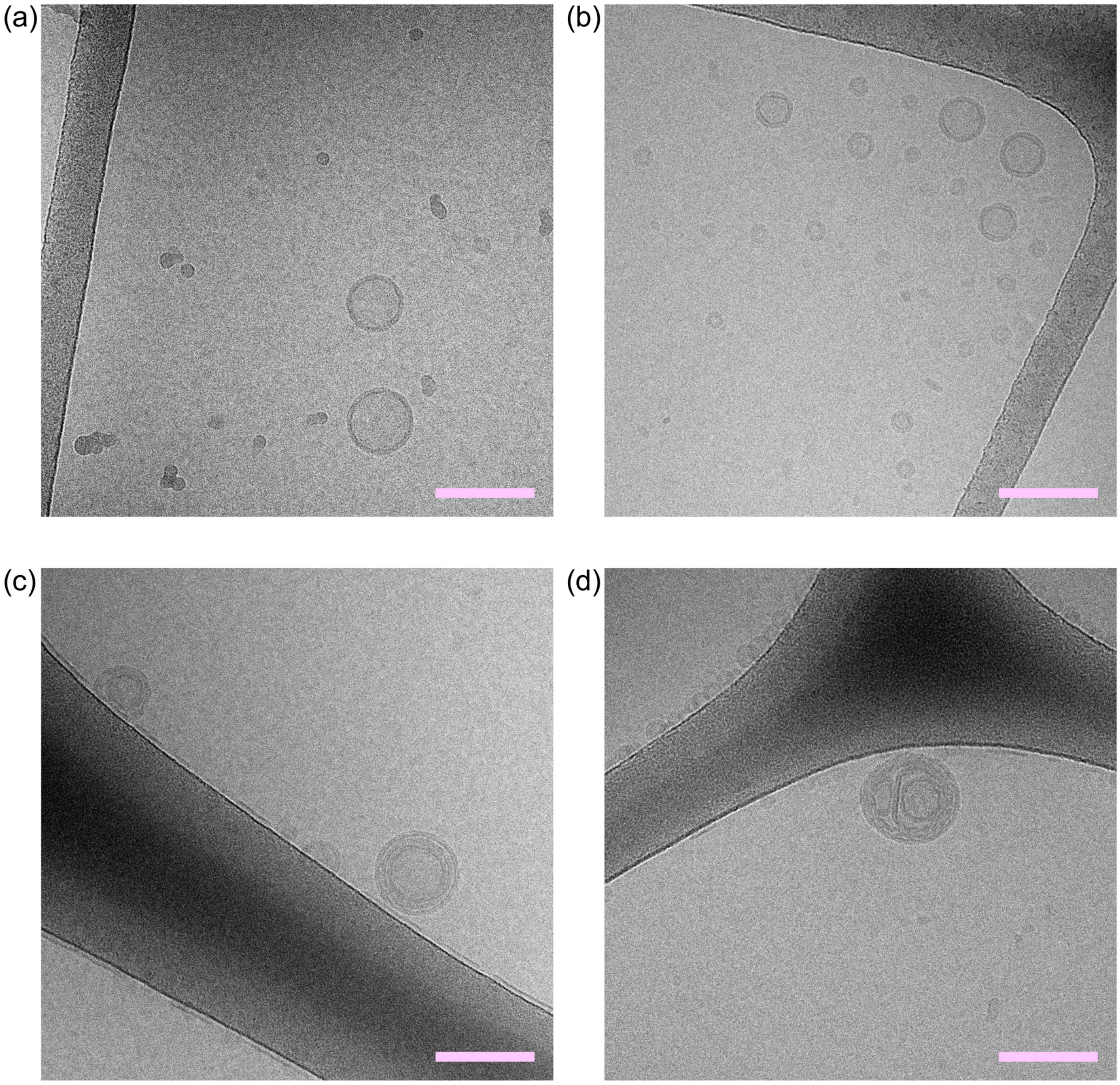


**Figure S4.** (a) And (b), additional cryo-TEM images of POPC:POPS 8:2 vesicles from the same sample as in Figure 2b in the main manuscript. (c) And (d), additional cryo-TEM images of POPC vesicles from the same sample as in Figure 2c. The scale bars show 100 nm.

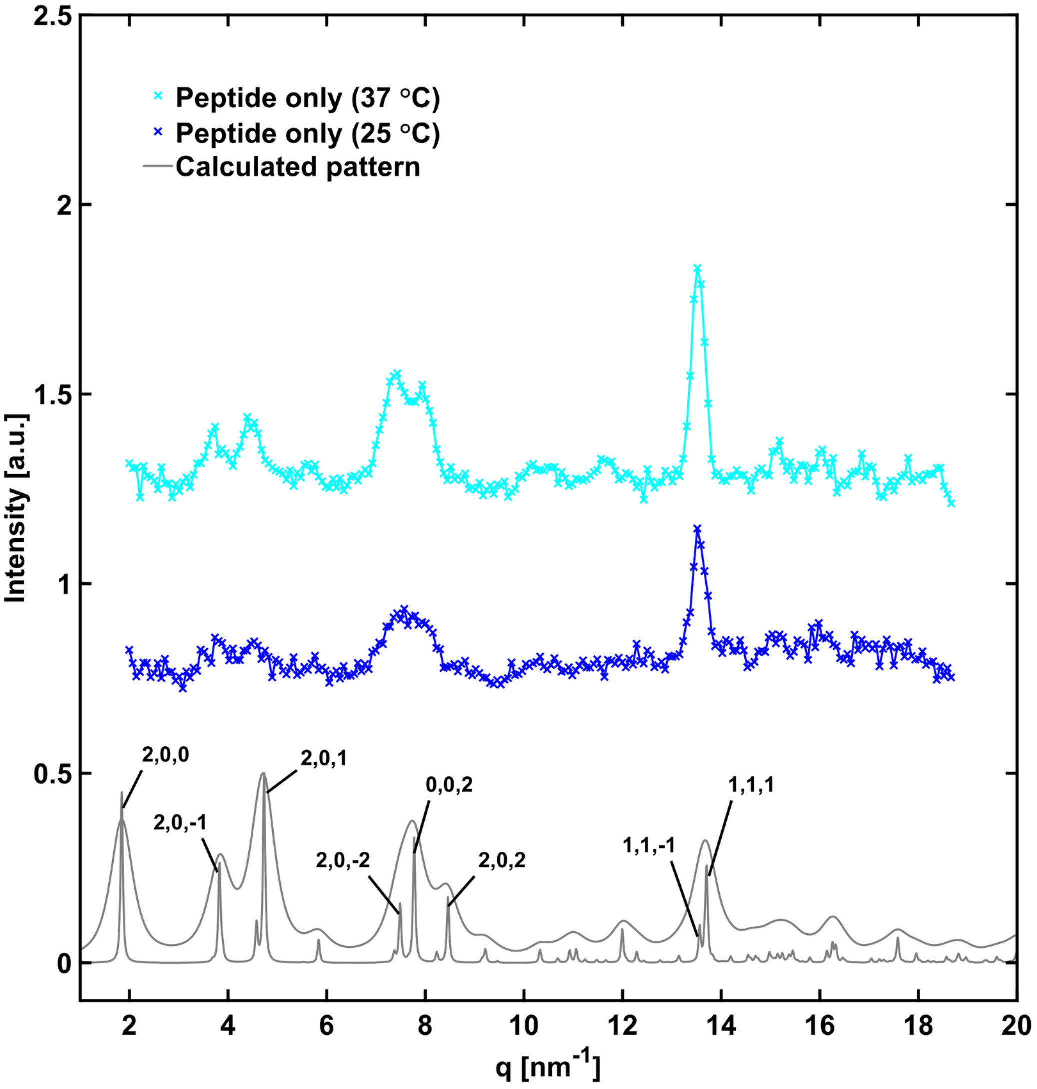


**Figure S5.** Comparison of wide angle X-ray scattering (WAXS) patterns for fibrils of peptide alone at room temperature (25 °C) and 37 °C (the same samples as in Figure 3 and 7a in the main manuscript, respectively). At the bottom (gray) is shown a calculated powder diffraction pattern based on the cryogenic electron diffraction NACore crystal structure determined by Rodriguez et al. (2015) (PDB ID: 4RIL). The powder diffraction pattern was calculated using Mercury 3.10 (The Cambridge Crystallographic Data Center, CCDC) (Macrae et al., 2008) and is shown together with corresponding miller indices of the main diffraction peaks.

**Table S1.** Details of the models used to fit the scattering data in the main manuscript. The table shows the model types implemented from SasView, and the parameters used.

| **Peptide alone (fit 1)** | | | **Peptide alone (fit 2)** | | |
|---|---|---|---|---|---|
| **Model type** | **Parameters** | | **Model type** | **Parameters** | |
| Lamellar | Median* layer thickness (nm) | 6.5 | Mass-fractal | Median* building block radius (nm) | 4.3 |
| | Layer thickness spread* | 1.5 | | Building block radius spread* | 1.5 |
| | Layer sld ($10^{-6}$ Å$^{-2}$) | 1 | | Fractal dimension | 2.35 |
| | Solvent sld ($10^{-6}$ Å$^{-2}$) | 0 | | Cutoff length (nm) | 10000 |

| **POPC:POPS 8:2 alone** | | |
|---|---|---|
| **Model type** | **Parameters** | |
| Core-multi-shell | Median* core radius (nm) | 15 |
| | Core radius spread* | 2.0 |
| | Core sld ($10^{-6}$ Å$^{-2}$) | 9.5 |
| | Shell 1 thickness (nm) | 0.75 |
| | Shell 1 sld ($10^{-6}$ Å$^{-2}$) | 13.5 |
| | Shell 2 thickness (nm) | 3 |
| | Shell 2 sld ($10^{-6}$ Å$^{-2}$) | 8.0 |
| | Shell 3 thickness (nm) | 0.75 |
| | Shell 3 sld ($10^{-6}$ Å$^{-2}$) | 13.5 |
| | Solvent sld ($10^{-6}$ Å$^{-2}$) | 9.5 |

| **POPC alone** | | | | | |
|---|---|---|---|---|---|
| **90 %** | | | **10 %** | | |
| **Model type** | **Parameters** | | **Model type** | **Parameters** | |
| Core-multi-shell | Median* core radius (nm) | 10 | Core-multi-shell | Median* core radius (nm) | 10 |
| | Core radius spread* | 3.0 | | Core radius spread* | 3.0 |
| | Core sld ($10^{-6}$ Å$^{-2}$) | 9.5 | | Core sld ($10^{-6}$ Å$^{-2}$) | 9.5 |
| | Shell 1 thickness (nm) | 0.75 | | Shell 1 thickness (nm) | 0.75 |
| | Shell 1 sld ($10^{-6}$ Å$^{-2}$) | 13.5 | | Shell 1 sld ($10^{-6}$ Å$^{-2}$) | 13.5 |
| | Shell 2 thickness (nm) | 3 | | Shell 2 thickness (nm) | 3 |
| | Shell 2 sld ($10^{-6}$ Å$^{-2}$) | 8.0 | | Shell 2 sld ($10^{-6}$ Å$^{-2}$) | 8.0 |
| | Shell 3 thickness (nm) | 0.75 | | Shell 3 thickness (nm) | 0.75 |
| | Shell 3 sld ($10^{-6}$ Å$^{-2}$) | 13.5 | | Shell 3 sld ($10^{-6}$ Å$^{-2}$) | 13.5 |
| | Solvent sld ($10^{-6}$ Å$^{-2}$) | 9.5 | | Shell 4 thickness (nm) | 2 |
| | | | | Shell 4 sld ($10^{-6}$ Å$^{-2}$) | 9.5 |
| | | | | Shell 5 thickness (nm) | 0.75 |
| | | | | Shell 5 sld ($10^{-6}$ Å$^{-2}$) | 13.5 |
| | | | | Shell 6 thickness (nm) | 3 |
| | | | | Shell 6 sld ($10^{-6}$ Å$^{-2}$) | 8.0 |
| | | | | Shell 7 thickness (nm) | 0.75 |
| | | | | Shell 7 sld ($10^{-6}$ Å$^{-2}$) | 13.5 |
| | | | | Solvent sld ($10^{-6}$ Å$^{-2}$) | 9.5 |

***Refers to the median ($e^{\mu}$) and geometric standard deviation ($e^{\sigma}$) of a lognormal probability distribution**

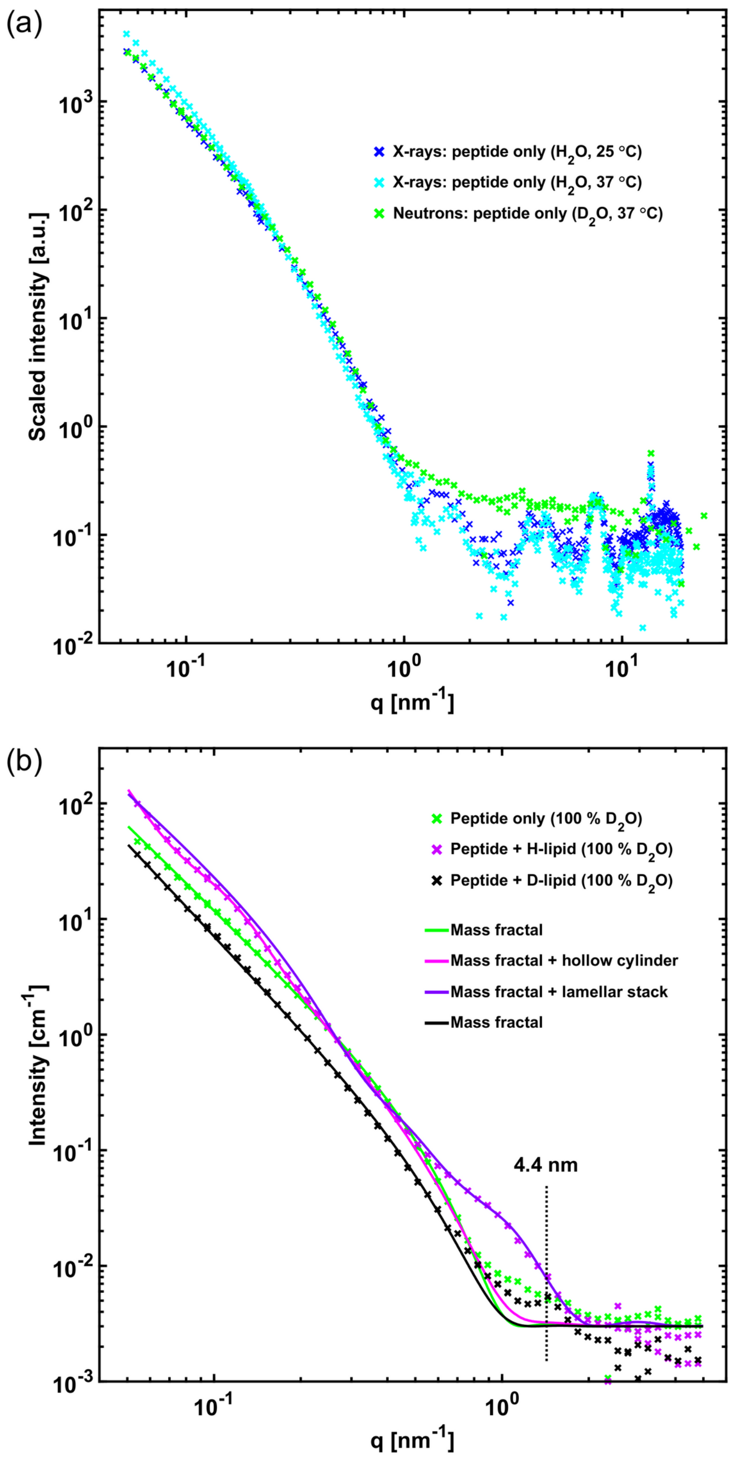


**Figure S6.** (a) Comparison of X-ray and neutron scattering data for NACore fibrils alone (formed and measured at 25 °C or 37 °C, with $H_2O$ or $D_2O$ as solvent). The scaled curves overlap at low q-values, except for a slightly steeper slope for the X-ray sample in $H_2O$ at 37 °C. At high q-values diffraction peaks can be resolved with X-rays, but they were not strong enough to be resolved with neutrons. (b) Neutron scattering data of NACore without and with DMPC:DMPS. "H-lipid" denotes undeuterated lipids, whereas "D-lipid" denotes lipids with deuterated acyl chains. The various fittings are consistent with the presence of multilamellar helical tubes like those shown in Figure S7 for the samples with peptide + H-lipid or D-lipid.

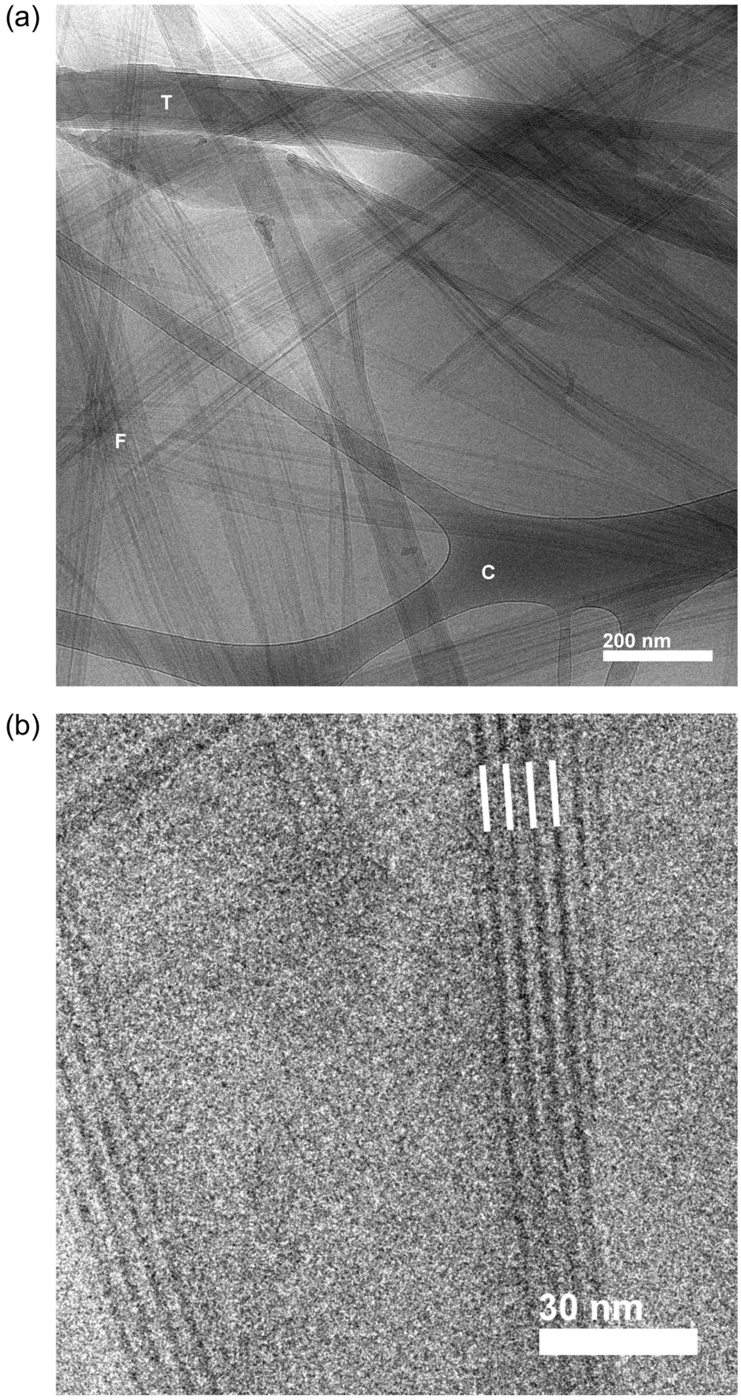


**Figure S7.** NACore together with DMPC:DMPS. (a) Example of multilamellar helical tubes found together with fibrils in the sample. "C" denotes the cryo-TEM carbon grid, "F" denotes fibrils, and "T" denotes multilamellar helical tubes. (b) Closeup of the wall of a multilamellar helical tube, revealing a repeat distance of 4.4 nm between the layers.

S1. Neutron scattering and cryo-TEM experiments with peptide and DMPC:DMPS

For the neutron scattering experiments, the samples were prepared using the same procedure as for X-ray scattering, except that the solvent used was $D_2O$ instead of $H_2O$, that fibrillation (and measurements) were performed at 37 °C instead of room temperature, and that equipment of other brands were used. The phospholipids used were either DMPC:DMPS 8:2 ("H-lipid") or DMPC-D54:DMPS-D54 8:2 ("D-lipid") which are the same lipid species as DMPC:DMPS but with fully deuterated acyl chains. Measurements were performed at the TAIKAN (BL-15) time of flight based small and wide angle neutron scattering instrument (Takata et al., 2015) located at the spallation neutron source at the Material and Life Science Experimental Facility (MLF) at the Japan Proton Accelerator Research Complex (J-PARC) in the Ibaraki Prefecture of Japan (proposal No. 2018A0112). Each sample was measured in a quartz cuvette with a 1 mm path length, with a total exposure time of 3 hours. The $D_2O$ solvent was also measured separately in a similar way, and its scattering subtracted from the scattering profiles of the samples before plotting. Fittings were performed using standard scattering form factor models found in the SasView software package (http://www.sasview.org/).

Samples for cryo-TEM of peptide and DMPC:DMPS 8:2 were prepared in a similar way as for the scattering experiments, except that the samples were diluted by a factor of 100 in buffer right before the vitrification step for the cryo-TEM imaging, and $H_2O$ was the solvent. After vitrification, the cryo-TEM imaging was performed in the same way as described in the main manuscript.

Figure S6 shows neutron scattering data of NACore without and with DMPC:DMPS. "H-lipid" denotes undeuterated lipids, whereas "D-lipid" denotes lipids with deuterated acyl chains. Undeuterated lipids have a high contrast for the scattering of neutrons in $D_2O$. Consequently, the scattering profile of peptide + H-lipids shows substantial contribution from both the peptide and lipid component over the entire q-range. Deuterated lipids have a much smaller average contrast for neutron scattering in $D_2O$, and make a negligible contribution to the scattering profile for the peptide + D-lipid sample at low q-values (this is similar to the situation in the X-ray scattering experiments, where the average scattering contrast for the lipid component is small). In Figure S6b the scattering profile of peptide + H-lipid could be fitted, at low q-values, as a linear combination of the peptide scattering and the calculated scattering of hollow core-shell cylinders with a mean internal diameter of 36 nm and a mean wall thickness of 18 nm. At higher q-values structures at smaller length scales become important, and the scattering profile of peptide + D-lipid was fitted as a linear combination of the peptide scattering and a lamellar stack with 4 layers, 2.9 nm layer thickness, and a mean repeat distance of 4.4 nm (lamellar stack paracrystal). The small peak in the scattering profile for peptide + D-lipids is shown to be at a q-value corresponding to a 4.4 nm periodicity in real space ($q = 1.43\ nm^{-1}$). These structures and dimensions are consistent with the presence of multilamellar helical tubes similar to those shown in the cryo-TEM images in Figure S7, in the samples with co-incubated peptide and lipid.

S2. Estimation of multivalent cation concentration by elemental analysis

For elemental analysis freeze-dried NACore peptide was vigorously dispersed in $H_2O$ (1 h at 700 rpm in an orbital vortexer) at a concentration of 1 mg/ml followed by centrifugation for 1 h at 15000 rcf at 20 °C. The resulting supernatant was analyzed by inductively coupled plasma optical emission spectrometry (ICP-OES). Duplicate samples were analyzed and the results indicated the presence of 2.08 and 2.09 µg/ml Ca, respectively for each sample, as well as 0.67 and 0.68 µg/ml Mg. This corresponds to estimated concentrations of approximately 16 µM $Ca^{2+}$ and 8 µM $Mg^{2+}$ in

the solution at the peptide concentration used for the scattering experiments (0.3 mg/ml). The analysis was performed by the Inorganic analysis laboratory at the Department of Biology at Lund University.

References

Macrae, C.F., Bruno, I.J., Chisholm, J.A., Edgington, P.R., McCabe, P., Pidcock, E., Rodriguez-Monge, L., Taylor, R., van de Streek, J., Wood, P.A. (2008). Mercury CSD 2.0 - new features for the visualization and investigation of crystal structures. Journal of Applied Crystallography 41:466–470. https://doi.org/10.1107/S0021889807067908

Takata, S., Suzuki, J., Shinohara, T., Oku, T., Tominaga, T., Ohishi, K., Iwase, H., Nakatani, T., Inamura, Y., Ito, T., Suzuya, K., Aizawa, K., Arai, M., Otomo, T., Sugiyama, M. (2015). The Design and q Resolution of the Small and Wide Angle Neutron Scattering Instrument (TAIKAN) in J-PARC. JPS Conference Proceedings 8:036020. https://doi.org/10.7566/JPSCP.8.036020
