# Supplementary figures and images for: NACore Amyloid Formation in the Presence of Phospholipids
Source: Front Physiol. 2020 Dec 18;11:592117. doi: 10.3389/fphys.2020.592117 (PMC7775532; doi:10.3389/fphys.2020.592117)

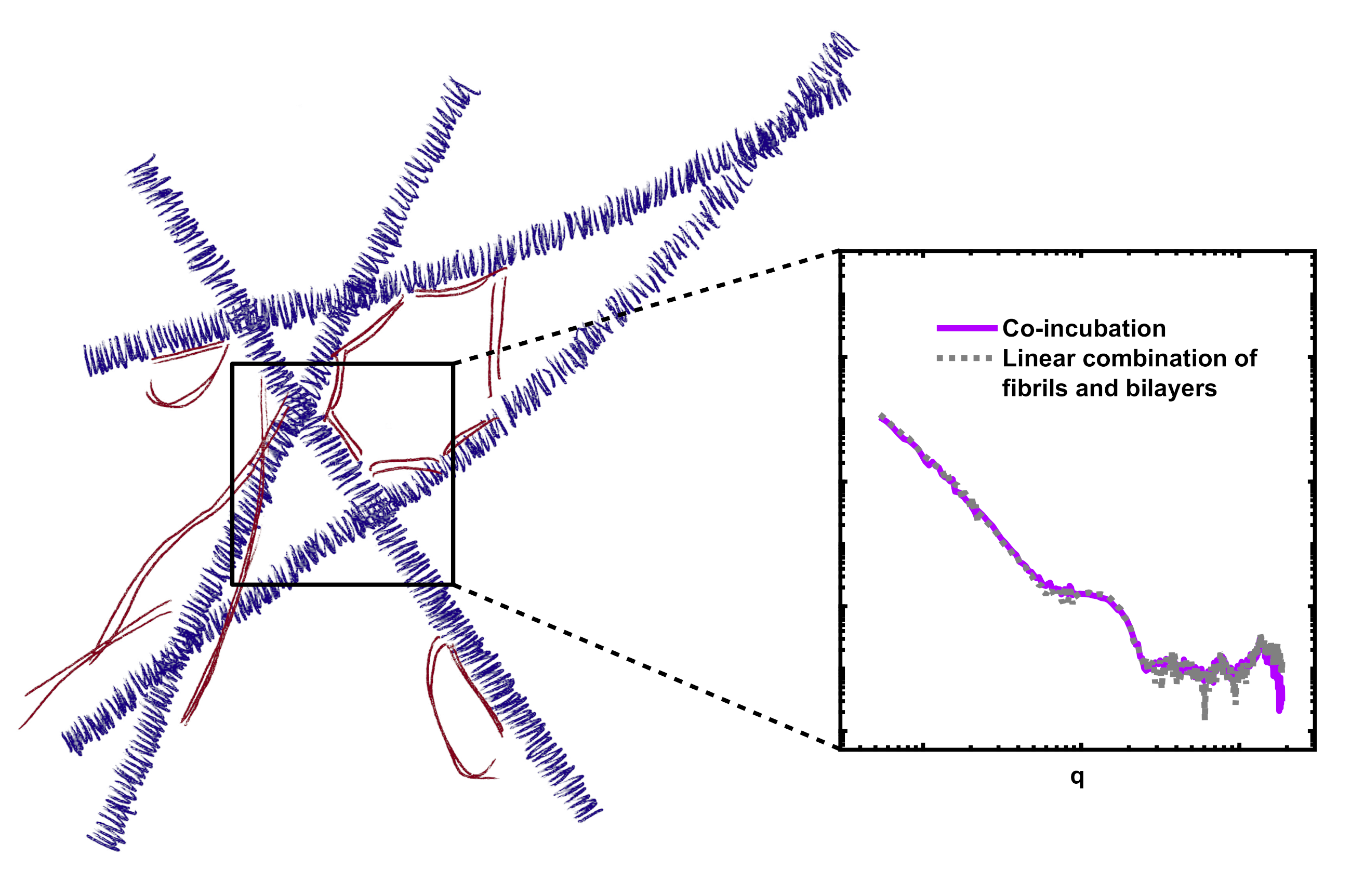

Supplement: Supplementary file 2 [file Image_1.TIF]
